# Single-Molecule Imaging of Wood Xylans on Surfaces and Their Interaction with GH11 Xylanase

**DOI:** 10.1021/acs.biomac.4c01446

**Published:** 2025-02-27

**Authors:** Jana B. Schaubeder, Christian Ganser, Chonnipa Palasingh, Manuel Eibinger, Tiina Nypelö, Takayuki Uchihashi, Stefan Spirk

**Affiliations:** † Graz University of Technology, Institute of Bioproducts and Paper Technology, Inffeldgasse 23, 8010 Graz, Austria; ‡ National Institutes of Natural Sciences, Exploratory Research Center on Life and Living Systems, 5-1 Higashiyama, Myodaiji, 444-8787 Okazaki, Japan; § Department of Bioproducts and Biosystems, Aalto University, 00076 Aalto, Finland; ∥ Institute of Biotechnology and Biochemical Engineering, Graz University of Technology, 8010 Graz, Austria; ⊥ Department of Chemistry and Chemical Engineering, Chalmers University of Technology, 41296 Gothenburg, Sweden; # Wallenberg Wood Science Center, Chalmers University of Technology, 41296 Gothenburg, Sweden; ∇ Department of Physics, Nagoya University, Chikusa-ku, Furo-cho, 464-8602 Nagoya, Japan

## Abstract

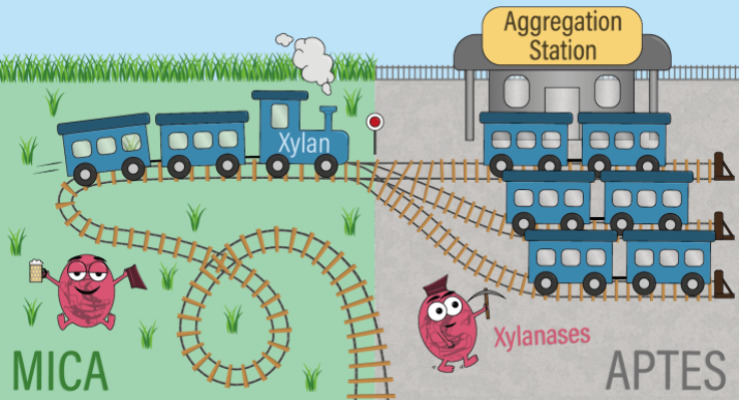

The knowledge of the molecular properties and arrangements of biopolymers in both solid and solution state are essential in the design of sustainable materials and biomedicine as they are decisive for mechanical strength, flexibility, and biodegradability. However, the structure of most biopolymers at charged interfaces can vary considerably, and their time-dependent visualization in liquid-state still remains challenging. In this work, we employed high-speed atomic force microscopy (HS-AFM) to visualize single xylan macromolecules from alkali-extracted birch and beechwood. On negatively charged mica surfaces, they appeared as individual macromolecules but assembled into aggregates on 3-aminopropyltriethoxysilane (APTES) surfaces (AP-mica). Hence, we further investigated the susceptibility to enzymatic degradation using an endoxylanase, which showed that the individual xylan macromolecules remained intact, while larger assemblies on AP-mica degraded over time. We demonstrate that HS-AFM is a powerful tool for understanding the molecular properties and degradation mechanisms of biopolymers. Moreover, by identifying alignment-dependent binding sites, strategies can be developed to ensure the biodegradability of composite materials by intelligent interface design.

## Introduction

Knowing the detailed molecular arrangements of biopolymers is crucial for understanding their functional properties, such as mechanical strength, flexibility, and enzymatic degradation. However, their arrangements depend also on the substrate to which they are bound. Xylan, the major hemicellulose component in plant cell walls, plays a critical role in the structural integrity and biochemical functionality of plants. Hence, numerous studies focus on the detailed interactions between xylan and cellulose to elucidate its role in the plant cell wall.^1−4^ It is, besides lignin, an underutilized resource with limited industrial applications so far. A main approach to valorizing xylans into products is to use enzymatic conversions. A challenge in these conversions is that there are different xylan conformations, which show different susceptibility to enzymes, as the conformation can determine the role of xylan in the plant.^1^ The tightly bound two-fold helical xylan is more resistant to enzymatic breakdown, which in turn impacts biomass conversion strategies by targeting specific xylan conformations for enzymatic treatment.^5,6^

Despite extensive molecular dynamics simulations and solid-state NMR spectroscopic results suggesting the existence of xylan in 2-fold or 3-fold helices,^1,2,7−9^ direct visualization of xylan as single macromolecules has remained elusive. In the past few decades, atomic force microscopy (AFM) has evolved rapidly, introducing new imaging modes to improve its versatility, speed, and quantitative analysis in polymer science. Advances in single-molecule force spectroscopy enable the determination of mechanical properties (e.g., elasticity and elongation) of individual proteins or polymer macromolecules, but also the investigation of polymerization processes in situ (e.g., kinetics and polymer growth mechanisms).^10−12^ Further advances such as high-speed atomic force microscopy (HS-AFM) allow for direct observation of dynamic molecular processes of biopolymers with a high spatiotemporal resolution. This enables the investigation of dynamic rearrangements of individual polymer macromolecules in different environments.^13,14^ However, the structure of most biopolymers is not rigid and varies with the conditions (temperature, pH) and surface charge of the substrate.^15^ Investigating these substrate-dependent changes in xylan assembly can open the way for customizing biocomposites and fine-tuning their properties. In biocomposites, it is crucial to find a compromise between strong adhesions between the natural fibers and the polymer matrix while maintaining biodegradability. To ensure biodegradability, the fibers need to remain accessible to enzymes, which requires a specific molecular arrangement that facilitates the formation of an enzyme–substrate complex.

Here, we used HS-AFM to directly visualize individual xylan macromolecules extracted from birchwood and beechwood. These xylan macromolecules adopted different conformations, depending on the substrate surface. Looking into biodegradability of these arrangements, we investigated the degradation mechanisms of xylan using an endoxylanase from the glycoside hydrolase (GH) family 11, which specifically targets xylan backbones. Depending on the conformation of the xylan on the different surfaces, the xylan was susceptible to enzymatic cleavage or not. This difference in susceptibility emphasizes the importance of molecular arrangements in determining the degradation kinetics of biopolymers. By identifying binding sites that depend on the arrangement and hence on the substrate properties, we can develop strategies to control the biodegradability of materials.

## Materials and Methods

### Materials

Xylan isolated from beechwood with a glucuronic acid *O*-methyl substitution of 12 mol % was purchased from Megazyme Ltd. (Ireland). Xylan isolated from birchwood with a glucuronic acid *O*-methyl substitution of 10 mol %, sodium phosphate monobasic dehydrate (purum p.a., crystallized, ≥ 99.0%), and sodium phosphate dibasic dehydrate (BioUltra, ≥ 99.0%) were purchased from Sigma-Aldrich (USA). Endo-1,4-β-d-xylanase from *Neocallimastix patriciarum* (GH11) was purchased from Megazyme Ltd. 3-Aminopropyltriethoxysilane (APTES, serial number KBE-093) was purchased from Shin-Etsu (Japan). Dimethyl sulfoxide (DMSO, purity ≥99.9%, serial number 472301–100 ML) was purchased from Sigma-Aldrich (USA). All chemicals were used as received. Mica was purchased from Furuuchi Chemical (Japan). For the preparation of the buffer, Milli-Q water (resistivity = 18.2 MΩ cm) from a Millipore water purification system (Millipore, USA) was used.

### Xylan Characterization

The monosaccharide compositions were determined by a two-step acid hydrolysis using 72% sulfuric acid at 30 °C followed by 4% sulfuric acid at 125 °C.^16^ The hydrolyzed xylan was filtered with a 0.2 μm PDVF filter to remove the acid-insoluble fraction, and fucose (200 mg mL^–1^) was used as standard and added to 1 mL of filtrate, which was then diluted 50 times with water. The monosaccharides were analyzed with high-performance anion exchange chromatography with pulsed amperometric detection (HPAEC-PAD) (Dionex ICS-3000 equipped with a CarboPac PA1 analysis column; Dionex Corporation, USA). NaOH/NaAc and NaOH were used as eluents. The molecular weights of the xylans were determined by size exclusion chromatography (SEC) using 0.01 M LiBr in a DMSO-based eluent. Approximately 4 mg of xylan was first swollen in 30 μL ultrapure water overnight, followed by dissolution in 2 mL of eluent for several days at room temperature and filtration through a 0.45 μm PTFE syringe filter. 100 μL of filtered xylan solution was injected into the SEC system equipped with a Jordi xStream GPC column (Jordi Laboratories, MA, USA) and analyzed using refractive index (RI) and right-angle light scattering (RALS, 670 nm, 90°) detectors. The column temperature was 60 °C, the detector temperature was 40 °C, and the flow rate was 0.8 mL min^–1^. Five pullulan standards with known molar masses were run as calibrations to calculate the molar mass of the unknown samples.

### Preparations for HS-AFM Measurements

Xylan was dissolved in DMSO at a concentration of 10 g L^–1^ aided by activation with distilled water (2% v/v; swelling for several hours, then addition of DMSO). The xylan solution (1 μL) was then pipetted either on a clean mica surface or on AP-mica and fixed onto the sample holder. After 10 min, the mica surface was rinsed with ultrapure water by holding a tissue close to the liquid, making use of capillary forces. It was made sure that the surface did not dry during this process. This washing step with SPB served to remove unbound xylan, as well as DMSO residues, to avoid artifacts. The adsorption of APTES onto the mica surface was performed by pipetting 2 μL of APTES (0.1% in ultrapure water; prepared directly before the measurement) onto the mica surface and washed with ultrapure water after 3 min to remove any excess material, as described above, before the addition of the xylan solution. As APTES can polymerize in water, it is essential to dilute it just before use and limit the incubation time to achieve a flat monolayer of silane with low surface roughness.^17−20^ Surface modification with APTES is expected to create a uniformly positively charged surface as described in the literature.^21−24^ However, the charge of the actual surfaces was not determined in this study due to experimental limitations regarding sample size. Endo-1,4-β-xylanase (GH11, *N. patriciarum*) was diluted 1000-fold to 10 U mL^–1^ (12.5 μg mL^–1^) in 100 mM sodium phosphate buffer (SPB), pH 6. One unit of xylanase activity is defined as the amount of enzyme required to release one μmol of xylose-reducing sugar equivalents per minute from wheat arabinoxylan (Megazyme Ltd., Ireland).

### HS-AFM Measurements

HS-AFM measurements were conducted with a laboratory-built system.^25^ The sample is adsorbed on a mica disc glued to a glass rod which is fixed on the z-piezo. The sample performs the *x*-, *y*-, and *z*-motions during scanning, while the cantilever is stationary. All measurements were performed in tapping mode, with the cantilever oscillating close to its resonance frequency. Cantilevers were 9 μm long, 2 μm wide, with a spring constant between 0.1 and 0.3 N m^–1^, and a resonance frequency in aqueous solution of about 700 kHz. Using electron beam deposition, carbon tips were grown at the very end of the cantilever with an apex radius of about 2 nm. The free amplitude in tapping mode was about 2 nm, and the set-point was adjusted to 80% of the free amplitude. All HS-AFM measurements were done with the sample immersed in 100 mM SPB (pH 6). For xylan degradation, 2 μL of the 12.5 μg mL^–1^ xylanase solution was added during the measurement into 80 μL SPB (*c*_xylanase_ = 11.8 nM). To observe single xylanase, a clean mica surface was imaged while 2 μL of the 12.5 μg mL^–1^ xylanase solution was added into 80 μL SPB.

## Results and Discussion

**Figure 1:**
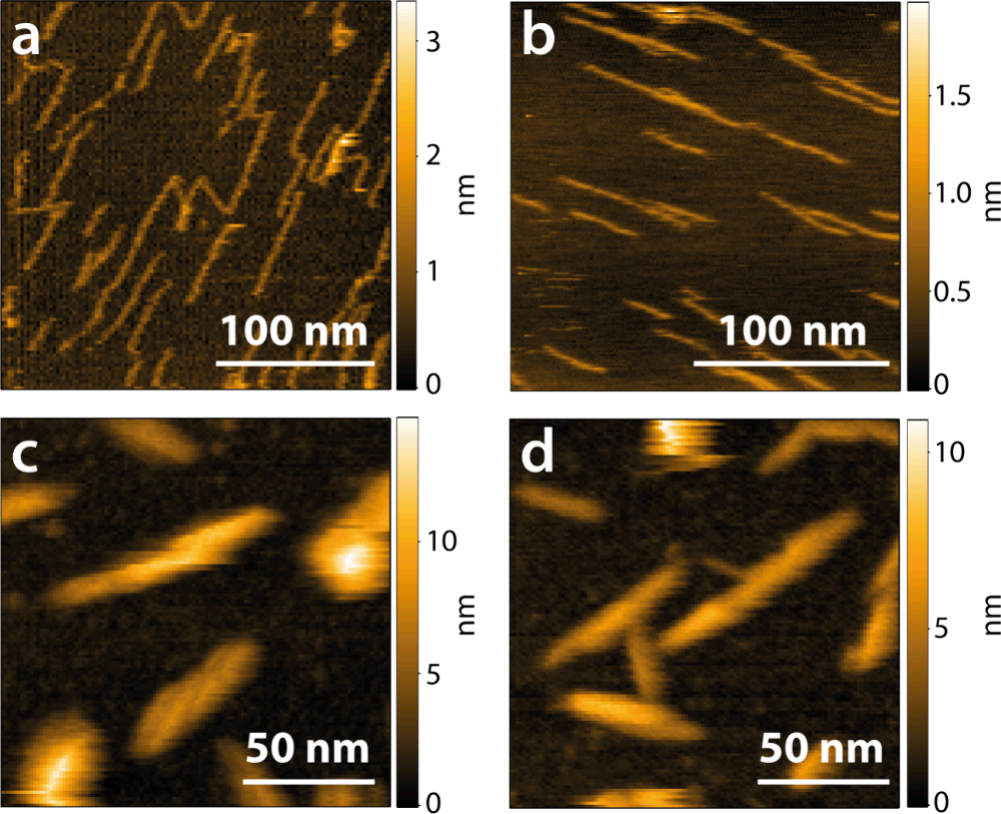
Individual macromolecules of (a) BIX and (b) BEX formed on mica. Larger assemblies of (c) BIX and (d) BEX formed on AP-mica.

We employed two xylans (from birchwood, BIX, and beechwood, BEX), which are similar in terms of xylose content (99 vs 96%) and weight-average molar mass *M*_w_ (19.4 kDa vs 23.8 kDa) (Table S1). The main differences between the two xylans are the methylglucuronic acid contents (10 vs 12%, for BIX and BEX, respectively) and that BIX has a broader molar mass distribution than BEX (*Đ* of 1.75 vs 1.3), indicating larger variations in the total chain length. The xylans were dissolved in DMSO (10 g L^–1^) using a preswelling procedure with distilled water.^26^ The solutions were then deposited on negatively charged mica surfaces and 3-aminopropyltriethoxysilane-treated mica (AP-mica) for several minutes, followed by a rinsing step to remove the reversibly bound material. Both xylans adsorbed in single chain configurations on the mica surface (Figure 1a,b), while larger assemblies were observed on the AP-mica surface (Figure 1c,d). AP-mica has been extensively used to immobilize DNA and proteins.^18−20^ In this method, developed by Lyubchenko et al.,^21^ APTES covalently binds to freshly cleaved mica resulting in a weakly cationic surface under aqueous conditions below the p*K*_a_ of the amino groups (p*K*_a_ 10.5).^17,22−24,27,28^ The surface roughness of the AP-mica increased compared to the mica surface about 3 times from 0.1 to 0.3 nm, which was already discussed in the literature (Figure S1).^27^ While this increase is significant in comparison to bare mica, which is atomically flat, the roughness increase cannot account for the aggregates, which were observed with xylan present. Moreover, the AP-mica surface shows a few oligomerized APTES units with a diameter of about 10 nm and a height of 1–1.5 nm (Figure S1c,d), none of which exhibit a morphology comparable to Figure 1c,d.

The heights of the BIX and BEX macromolecules were 0.74 ± 0.05 nm (average maximum height of 49 line profile measurements of individual macromolecules, see Figure S2a) and 0.75 ± 0.04 nm (average maximum height of 30 line profile measurements of individual macromolecules, see Figure S2a), respectively. The assumed height of a xylose unit, 0.5 nm, is less than the measured height. However, a monolayer of water could be present between the mica surface and the xylan macromolecules upon rinsing with buffer after adsorption. The diameter of a water molecule is approximately 0.28 nm,^29,30^ resulting in a total height of 0.78 nm, which agrees well with the measured height of 0.74 ± 0.05 nm. In addition, the methylglucuronic acid side chain substitutions at regular intervals of 8–9 units for BEX and 10 units for BIX further contribute to the overall height and could increase the height by about 0.5 nm every 4–5 nm. Although we do not have conclusive evidence to determine the precise reason for the observed discrepancy, these observations strongly suggest that we indeed observed individual xylan macromolecules with HS-AFM for both xylans (supporting HS-AFM images, see Figure S3a–f).

The average lengths of the BIX and BEX macromolecules were 62 ± 28 nm (BIX; avg of 141 individual macromolecules determined by automatic mapping, see Figure S2b) and 62 ± 20 nm (BEX; avg of 79 individual macromolecules determined by automatic mapping, see Figure S2b). The length determined for BIX (62 ± 28 nm) is in excellent agreement with the calculated average length for individual BIX macromolecules of 64.5 nm based on *M*_w_ and methylglucuronic acid content (116 unsubstituted and 13 substituted anhydroxylose units (AXU); calculations are given in detail in the electronic Supporting Information). The length determined for BEX (62 ± 20 nm) is slightly shorter than the calculated average length for individual BEX macromolecules of 76.5 nm based on the methylglucuronic acid content (135 unsubstituted and 18 substituted AXU). Although the macromolecules are adsorbed to the mica surface, some segments are flexible as they move during the measurement, probably induced by distortions of the AFM tip (Video S1: BIX, Video S2: BEX).

The average heights of the larger xylan assemblies on AP-mica surfaces were 6 ± 1 and 5 ± 1 nm (average maximum height of at least 15 line profile measurements) for BIX and BEX, respectively (supporting HS-AFM images Figure S3g–l). These assemblies have an average length of 61 ± 20 and 54 ± 17 nm and a width of 20 ± 3 and 14 ± 2 nm for the BIX and BEX, respectively. It should be noted that the lengths and widths are overestimated due to tip convolution effects. For the BIX, a clear twisting of the assemblies is visible; however, both xylan assemblies have a similar appearance with comparable dimensions. The BEX assemblies are slightly shorter and feature a smaller diameter, which might be related to the different methylglucuronic acid contents of the two xylans. Assuming a homogeneous substitution pattern, the distance between two methylglucuronic acid substitutions is shorter for the BEX with a higher amount of side chain substitutions (12 mol %) compared to the BIX (10 mol %). Hence, the substitution pattern could influence the dimensions of these assemblies.

**Figure 2:**
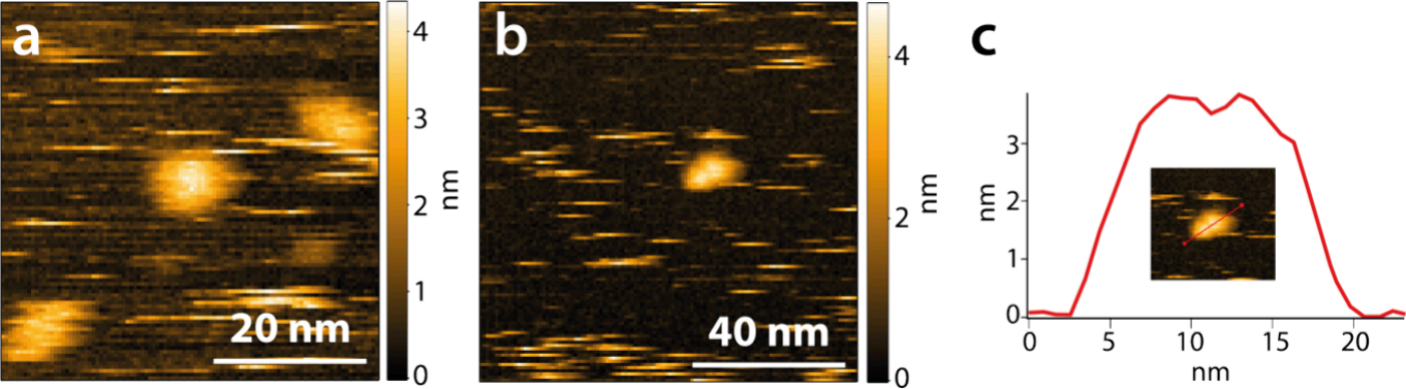
(a) Xylanases on the mica surface. (b) Xylanases on mica surface forming dimers, and (c) line profile of a xylanase dimer. The streaks in the HS-AFM images are caused by the rapidly moving xylanase molecules on the surface.

To investigate the enzymatic susceptibility of the two xylans in the different assemblies, a GH11 endoxylanase was used, which uses the methylglucuronic acid side chain substitution for docking and then cleaves the backbone one unit before the side chain substitution.^31^ The behavior of the xylanases on neat mica surfaces showed that there is no strong adsorption and that they can move rapidly on the surface, resulting in streaks in the HS-AFM images (Figure 2a, Video S3). A height of 3.2 ± 0.3 nm was determined for the xylanase (average maximum height of 18 line profile measurements of individual xylanases using 50 × 50 nm^2^ images). In general, the diameter of proteins can be estimated from their *M*_w_ using eq 1, assuming a partial specific volume of 0.73 cm^3^ g^–1^ and spherical shape.^32^ The factor of 0.132 includes the conversion of units as well as the conversion of the volume to the diameter of a sphere.
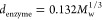
1With an *M*_w_ of 25,800 g mol^–1^, a diameter of 3.9 nm was calculated for the xylanase used, which corresponds well with the measured height of 3.2 ± 0.3 nm. Using structural data of the xylanase (PDB:3WP4), pseudo-AFM images were simulated using the hard sphere model, which resulted in a height between 4 and 5 nm (see Figure S4). These simulated images do not account for deformations caused by substrate interaction or tip deformation, marking the measured height of 3.2 ± 0.3 nm as reasonable. Moreover, the xylanase formed dimers (Figure 2b,c) and sometimes even trimers for short time intervals, as indicated by the larger aggregates formed in Video S3 at 19 s, 30 s, 50 s, and 92 s. This tendency to form dimers has not been observed for the xylanase used; however, it has been discussed in the literature for a similar xylanase.^33^

**Figure 3:**
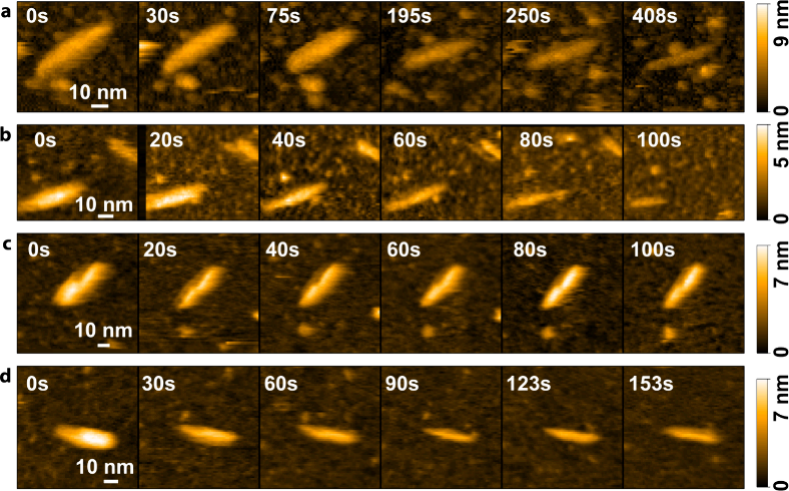
Degradation of xylan assemblies on AP-mica of (a, b) BIX and (c, d) BEX.

When the xylanases are added to the individual xylan macromolecules (Video S4: BIX+xylanase, Video S5: BEX+xylanase), they mainly move around the macromolecules. Chain cleavage is not observed over time, suggesting that the formation of an enzyme–substrate complex for cleavage is not successful under these conditions. The GH11 xylanase used has a catalytic cleft; therefore, it is assumed that this cleft cannot enclose the strongly adsorbed xylan macromolecules. When the xylanases are added to the larger xylan assemblies (Video S6: BIX assemblies, Video S7: BEX assemblies), the xylanases are not as visible at first due to the larger height of the xylan assemblies compared to the individual macromolecules. However, it appears that xylanases can penetrate these larger assemblies. This of course implies that cleavage within the xylan assemblies can occur, which is not directly evident from the HS-AFM measurements. However, over time, these xylan assemblies become significantly smaller (see Figure 3 as well as Videos S8 and S9 for BIX and BEX, respectively). The assemblies appear to break down stepwise on a larger scale, which could be caused by xylanases structurally weakening the larger assemblies (Video S10: Degradation of BEX assemblies). These structural changes could also be a result of distortions caused by the AFM tip. However, the xylan assemblies were stable over time without any xylanase added (Video S11: BIX assemblies, Video S12: BEX assemblies), indicating that the xylanases indeed partially degrade the xylan assemblies. In general, the used xylanase exhibits distributive rather than processive behavior, i.e., the enzyme binds, cleaves, and immediately dissociates from the substrate, which makes its visualization difficult. Rudimentary degradation kinetics can be calculated by measuring the length, width, and height of the aggregates shown in Figure 3, which will be referred to as BIX 1 (Figure 3a), BIX 2 (Figure 3b), BEX 1 (Figure 3c), and BEX 2 (Figure 3d) in the following. To focus on relative trends rather than reporting a less precise absolute value due to tip convolution effects, the volumes of the xylan assemblies were calculated and normalized. To obtain the kinetic parameters, we employed a simple model as described by Lee et al.,^34^ where the enzyme interacts with an immobilized substrate in the absence of bulk transport limitations, according to eq 2.
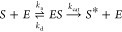
2Here, *E* represents the enzyme in solution, *S* represents the surface-bound substrate, *ES* represents the surface-bound enzyme–substrate complex, and S* represents the surface-bound product. The formation of the *ES* complex is governed by Langmuir adsorption/desorption rates *k*_a_ and *k*_d_, while the catalytic cleavage is characterized by the catalytic reaction rate *k*_cat_. Using numerical integration methods in Python, as described in detail in our earlier work,^35^ we obtained the reaction rate constants *k*_a_, *k*_d_, and *k*_cat_ for the degradation of the four xylan assemblies BIX 1, BIX 2, BEX 1, and BEX 2 (HS-AFM images illustrated in Figure 3; kinetic simulations of normalized volumes of xylan assemblies in Figure S5). Reaction rate constants for adsorption *k*_a_ 1.3 × 10^6^ – 2.3 × 10^6^ M^–1^ s^–1^, desorption *k*_d_ 0.02 – 4 s^–1^, and catalytic cleavage *k*_cat_ 121 – 152 s^–1^ were obtained (Table 1). However, given the limited data points and the inclusion of line profile measurement errors, the calculated values should be considered as estimates.

**Table 1:** Calculated Kinetic Parameters *k*_a_, *k*_d_, and *k*_cat_ for the Degradation of the Xylan Assemblies BIX 1, BIX 2, BEX 1, and BEX 2 with a GH11 Xylanase and Comparable Parameters Obtained for the Degradation of a 12 nm Thick BIX Thin Film Determined in Our Previous Work

xylan sample	*k*_a_ [M^–1^ s^–1^]	*k*_d_ [s^–1^]	*k*_cat_ [s^–1^]
BIX 1	1.3 × 10^6^	4	152
BIX 2	2.1 × 10^6^	0.9	140
BEX 1	2.0 × 10^6^	0.02	121
BEX 2	2.3 × 10^6^	0.5	136
BIX thin film^35^	2.8 × 10^5^	35	116

In our earlier work,^35^ we determined reaction rates of *k*_a_ 2.8 × 10^5^ M^–1^ s^–1^, *k*_d_ 35 s^–1^, and *k*_cat_ 116 s^–1^ (comparison of reaction rate constants is shown in Table 1) for the degradation of 12 nm thick BIX thin films by the used GH11 xylanase at the lowest flow rate (25 μL min^–1^), which is most comparable to the setup in this work. Compared to our previous work, a higher adsorption constant *k*_a_ was determined, indicating a higher affinity between the enzyme and substrate for the xylan assemblies. This can be explained by the better accessibility of the xylan assemblies compared to that of the xylan thin films, where *k*_a_ decreased significantly with increasing xylan film thickness. Similar to our previous work, the desorption constant *k*_d_ is about 7 orders of magnitude smaller than *k*_a_, indicating a strong affinity between the enzyme and substrate. Therefore, we do not discuss *k*_d_ here to avoid overinterpretation, because the data are not sensitive to *k*_d_, which can lead to a large deviation. Moreover, for distributive enzymes such as the xylanase used, adsorption is the rate-limiting step, hence *k*_a_ is more important than *k*_d_ for the overall reaction efficiency.^36^ Similar catalytic reaction rates *k*_cat_ of 121–152 s^–1^ were determined for the different xylan assemblies, which are slightly higher than for the degradation of the xylan thin film, indicating a faster conversion that can be explained again by the better accessibility of the xylan assemblies compared to the thin films.

Our results show that the adsorption of both xylans on mica surface occurs in accordance with established polymer adsorption theories,^37−40^ where a polymer adsorbs on solid surfaces from the solution state in three configurations: Loops, in which the polymer segments are not in direct contact with the surface; trains, in which the polymer segments are in direct contact with the surface; and tails, in which the terminal segments of the macromolecules are not in contact with the surface. This leads to a comprehensive classification of adsorbed polymers into strongly adsorbed polymers (trains) and loosely adsorbed polymers (loops).^37,39,41,42^ For polymer chains with more than 50 segments adsorbed from dilute solutions, trends were predicted that the adsorbed molecules adopt a flat conformation with more than 85% of the segments arranged in trains and less than 15% in loops, while tails hardly play a role. Thus, for the isolated polymer chains, the conformation is flat, most segments are arranged in trains, loops are rather short and tails are negligible.^37^ These observations revealed diverse molecular behavior, which determine the properties of bulk materials.^11,43^ However, there are only a few studies on individual biomacromolecules, including studies on polyhydroxybutyrate, pectin, and alginate.^44−46^ The focus of these studies, though, is mainly on the biopolymer itself without consideration of the substrate used.

In this paper, we show that xylan adsorbed in different configurations to negatively charged mica and weakly cationic AP-mica surfaces. Xylan strongly adsorbed to the mica surface according to established polymer adsorption theories by forming mainly trains with some tails and small amounts of loops (Videos S1 and S2). This indicates that the main parts of the individual xylan macromolecules adhere well to the mica surface, limiting their accessibility, which in turn could explain why the xylan macromolecules were not susceptible to enzymatic degradation by the xylanase. The methylglucuronic acid side groups have a p*K*_a_ value of approximately 3.3,^47^ meaning they are negatively charged in the used sodium phosphate buffer at pH 6. Consequently, it can be argued that they do not directly interact with the negatively charged mica surface but are oriented toward the solution. In contrast, when the weakly cationic AP-mica surface was used, larger xylan assemblies were formed but their formation is difficult to understand. Silberberg^40^ showed that concentration and solvent effects have a significant influence on the adsorption results obtained for isolated macromolecules. This influence should be excluded from our work, as we used the same xylan solution for adsorption on different surfaces. However, the polymer–surface energy interaction parameter (χ_S_) changes with the AP-mica surface. This parameter cannot normally be determined directly and in most cases is considered an adjustable parameter for data modeling.^48^ An adsorption behavior differing from the trains/loops/tails for a very low χ_S_ was also predicted by Scheutjens and Fleer,^37^ corresponding to a low polymer–surface interaction. However, a stronger polymer–surface interaction is expected for AP-mica, resulting from an increased electrostatic attraction between the positively charged AP-mica surface and the negatively charged methylglucuronic acid side groups. Although the electrostatic attraction on the weakly cationic AP-mica surface is not particularly strong, higher adsorption with a higher surface coverage would still be expected compared with the mica surface. Since this is not the case and xylan is a flexible macromolecule, we speculate that the xylan macromolecules fold over each other, creating ordered assemblies in which the negatively charged methylglucuronic acid residues are preferentially oriented toward the sides and in contact with the AP-mica surface or the buffer solution. A height of 5–6 nm was determined for these assemblies for both xylans, which would correspond to around 10 xylan macromolecules on top of each other. While xylans generally crystallize into hexagonal platelets,^49^ Kontturi and co-workers^50^ produced needle-like xylan nanocrystals with a height of 10 nm, which is in a comparable magnitude, but which were much longer (855 ± 40 nm). However, the xylan used in their study was basically linear; hence, no side chain substitutions disturb the formation of a crystalline-like assembly. Renneckar and co-workers^49^ reported that the side chain substitutions significantly affect the dimensions of these typically formed hexagonal xylan platelets. They concluded that the methylglucuronic acid side chains disrupt the close packing of the xylan chains in the crystal lattice and thus hinder the crystal growth. While the xylan platelets, also termed nanotiles, have a defined shape and size in scanning electron microscopy, they look rather fluffy when wet, with local disorders at the surface.^51^ Nevertheless, the recorded xylan nanotiles are much larger (one micron in diameter and 100 nm in thickness) than the ordered assemblies, which were observed by HS-AFM. Moreover, it has been shown that xylan crystals are susceptible to enzymatic degradation using an enzyme complex containing at least four different xylanases, first attacking the accessible edges of the crystals and then evolving to the centers.^52^

**Figure 4:**
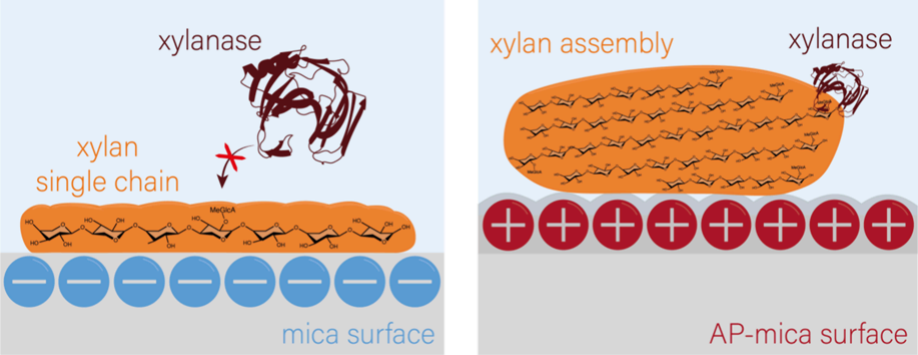
Schematic representation of the structure and conformation of xylan on a negatively charged mica surface (left) and a weakly cationic AP-mica surface (right). While the individual xylan macromolecules on mica are not accessible to a GH11 xylanase, the xylan assemblies formed on AP-mica can be enzymatically degraded over time.

A comprehensive overview of the combined effect of the substrate on the assembly and biodegradability of xylan is provided in Figure 4, which shows that the strong adhesion of the individual xylan macromolecules to the mica surface hinders the formation of an enzyme–substrate complex and thus also enzymatic degradation, while the larger xylan assemblies that form on AP-mica are susceptible to GH11 xylanase. For the individual xylan macromolecules, a xylan conformation is proposed in which the negatively charged methylglucuronic acid side groups point away from the negatively charged mica surface; nevertheless, the xylan remains inaccessible to the xylanase. For the xylan assemblies, a xylan conformation is proposed in which the methylglucuronic acid side chains point primarily toward the edges of the assemblies and toward the AP-mica, making the assemblies accessible to the xylanases. This difference in susceptibility emphasizes the importance of molecular arrangements, as well as the substrate charge, in determining the degradation kinetics of biopolymers. The results obtained would suggest that xylan also forms trains on cellulose, as it is negatively charged. We tried to verify this assumption by depositing cellulose on mica and then adsorbing xylan during the HS-AFM measurement. Unfortunately, this assumption could not be verified because of the structural similarity of cellulose and xylan in the wet state (Figure S6).

## Conclusions

We used HS-AFM to directly visualize individual xylan macromolecules extracted from birch and beechwood. These xylan chains showed different behaviors depending on the surface on which they were deposited. On a negatively charged mica surface, xylan was present as individual chains, while on weakly cationic AP-mica surfaces, it formed larger assemblies. When their biodegradability was assessed using a GH11 endoxylanase, it was observed that the individual xylan macromolecules on mica were resistant to enzymatic degradation, while the larger xylan assemblies on AP-mica were gradually degraded. This difference in susceptibility demonstrates how the molecular arrangement influences the rate of degradation of biopolymers. By identification of arrangement-dependent binding sites, strategies for enzymatic hydrolysis can be developed to control the biodegradability of materials and potentially enhance the valorization of biomass.

## Supporting Information Available

Detailed calculation of the length of the single xylan macromolecules and supporting HS-AFM images (PDF)
BIX single macromolecules with some flexible domains (MP4)
BEX single macromolecules with some flexible domains (MP4)
Xylanases on a mica surface moving freely (forming dimers at 19s, 30s, 50s, and 92s) (MP4)
Xylanases on individual BIX macromolecules (MP4)
Xylanases on individual BEX macromolecules (MP4)
Xylanases on BIX assemblies on AP-mica (MP4)
Xylanases on BEX assemblies on AP-mica (MP4)
Zooming into degradation of BIX assemblies on AP-mica (MP4)
Zooming into degradation of BEX assemblies on AP-mica (MP4)
Degradation of BEX assemblies on AP-mica (MP4)
Stable monitoring of birchwood xylan assemblies on AP-mica (MP4)
Stable monitoring of beechwood xylan assemblies on AP-mica (MP4)

## Abbreviations

APTES: 3-aminopropyltriethoxysilane
BEX: beechwood xylan
BIX: birchwood xylan
GH: glycoside hydrolase
HS-AFM: high-speed atomic force microscopy
AP-mica: APTES-modified mica
