# Supplementary material for: Single-Molecule Imaging of Wood Xylans on Surfaces and Their Interaction with GH11 Xylanase
Source: Biomacromolecules. 2025 Feb 27;26(3):1639–46. doi: 10.1021/acs.biomac.4c01446 (PMC11898079; doi:10.1021/acs.biomac.4c01446)
Supplement: Supplementary file 1 — bm4c01446_si_001.pdf [file bm4c01446_si_001.pdf]

Electronic Supporting Information

Single-molecule imaging of wood xylans on surfaces and their interaction with GH11 xylanase

Jana B. Schaubeder, Christian Ganser*, Chonnipa Palasingh, Manuel Eibinger, Tiina Nypelö, Takayuki Uchihashi, Stefan Spirk**

1. Detailed characterization of the two xylans

Table S1. Carbohydrate composition, molecular weights, polydispersity index and uronic acid side chain substitution of the used birchwood and beechwood xylans.

	Carbohydrate composition (relative)						M_w	PDI	Uronic acid substitution
	Xyl	Glu	Gal	Ara	Man	Rha	[g mol^{-1}]		(mol%)
Birchwood xylan	99	0	<1	<1	0	<1	19 400	1.75	10
Beechwood xylan	96	<2	<2	<1	0	0	23 800	1.3	12

2. Calculations using the molecular weights and the given substitution pattern

The actual length of a single xylan chain can be estimated using the molecular mass and the given substitution pattern, assuming a width (across the xylan ring) of 0.5 nm and a height (along the xylan ring) of 0.5 nm. For both xylans, an unsubstituted unit (x) has a molecular mass of 132 g mol^{-1}, while a substituted unit (y) has a mass of 131 g mol^{-1} + the methylglucuronic acid side group with a molecular mass of 191 g mol^{-1}. The stretched backbone of the xylan chain then equals the unsubstituted units x plus the substituted units y.

For the beechwood xylan a substitution of 12 mol% was given, as well as a molecular mass of 23,800 g mol^{-1}, which leads to Eqs. 1-2.

$$132\frac{g}{mol}\,x + 131\frac{g}{mol}\,y + 191\frac{g}{mol}\,y = 23\,800\frac{g}{mol} \qquad (1)$$

$$y = 0.12\,(x + y) \qquad (2)$$

By solving Eqs. 1 & 2, the beechwood xylan consists of 135 unsubstituted units and 18 substituted units, which gives a xylan chain backbone consisting of 153 units, corresponding to an actual length of **76.5 nm**.

For the birchwood xylan a substitution of 10 mol% was given, as well as a molecular mass of 19,400 g mol^{-1}, which leads to Eqs. 3 & 4.

$$132\frac{g}{mol}\,x + 131\frac{g}{mol}\,y + 191\frac{g}{mol}\,y = 19\,400\frac{g}{mol} \qquad (3)$$

$$y = 0.10\,(x + y) \qquad (4)$$

By solving Eqs. 3 & 4 the birchwood xylan consists of 116 unsubstituted units and 13 substituted units, which results of a xylan single chain backbone of 129 units, corresponding to an actual length of **64.5 nm**.

Of course the molecular weight of the xylans is a distribution, hence the molecular mass and therefore also the chain length vary, however a good estimate is obtained by the calculations. The PDI is neglected in these assumptions.

3. Substrate characterization by HS-AFM

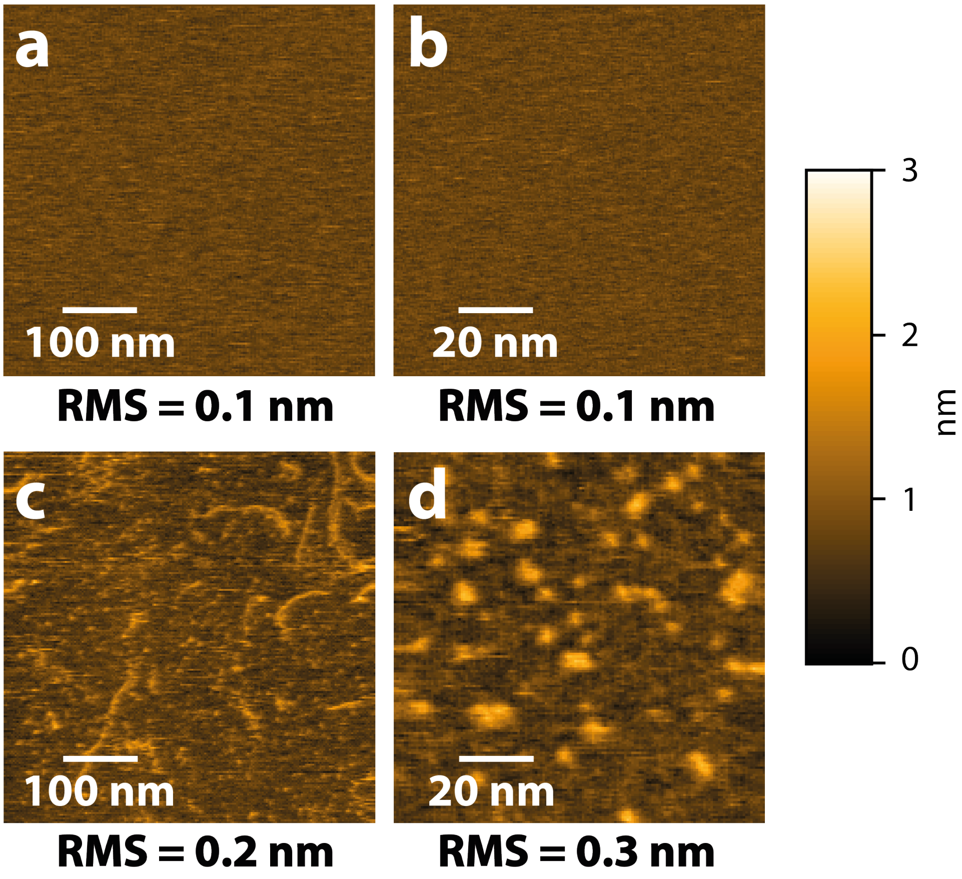

Fig. S1: AFM topography images of a,b) clean mica surface and c,d) AP-mica surface in SPB.

4. Distributions of xylan single macromolecule heights and lengths

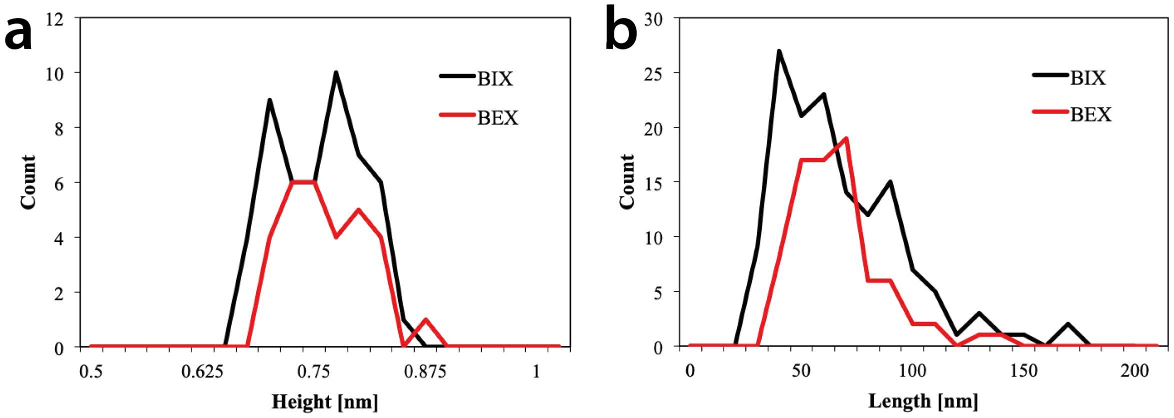

Fig. S2: a) Height distribution by line profile analysis of the maximal height of individual BIX and BEX macromolecules. b) Length distribution determined by automatic mapping of individual BIX and BEX xylan macromolecules.

5. Supporting HS-AFM images

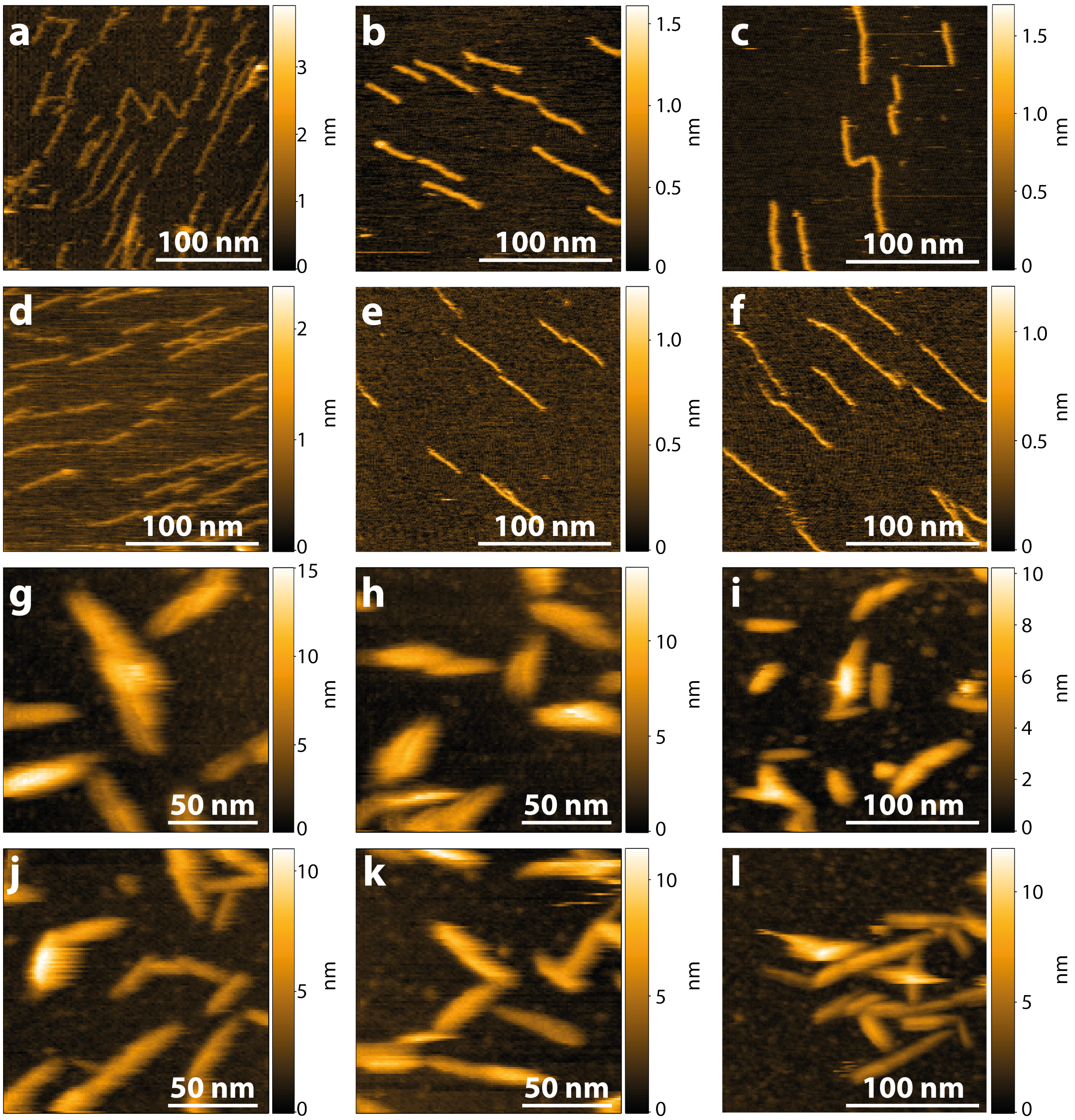

Fig. S3: Supporting HS-AFM images of a-c) BIX macromolecules on mica, d-f) BEX macromolecules on mica, g-i) BIX assemblies on APTES, and j-l) BEX assemblies on APTES.

6. Pseudo-AFM images of xylanase

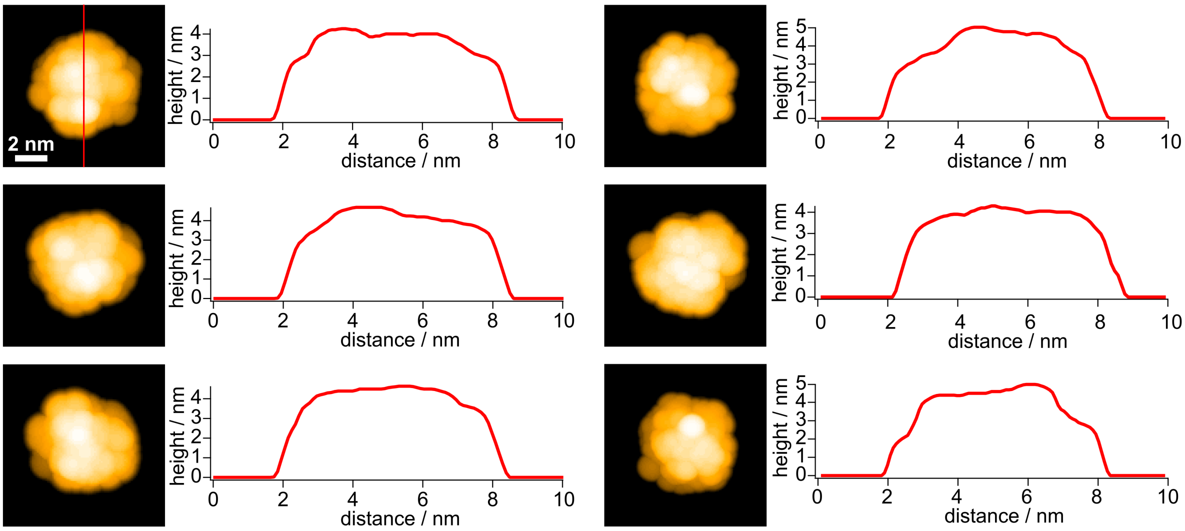

Fig. S4: Pseudo-AFM images of xylanase (PDB: 3WP4) at different orientations and corresponding height profiles (profile indicated by the red line on the top left). The simulation was performed by assuming rigid body interaction of a conical tip (10° opening angle) with a spherical cap (1 nm apex radius) with the molecular structure of xylanase.

7. Xylan degradation kinetics

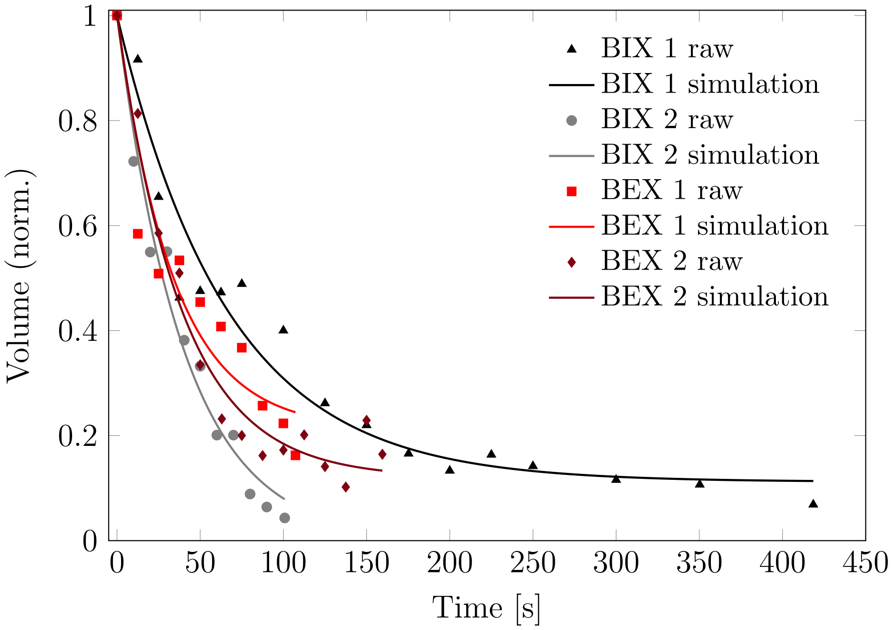

Fig. S5: Raw data and kinetic simulations of the volume decrease of xylan assemblies shown in Fig. 3 a) BIX 1, b) BIX 2, c) BEX 1, and d) BEX 2.

8. Xylan on Cellulose

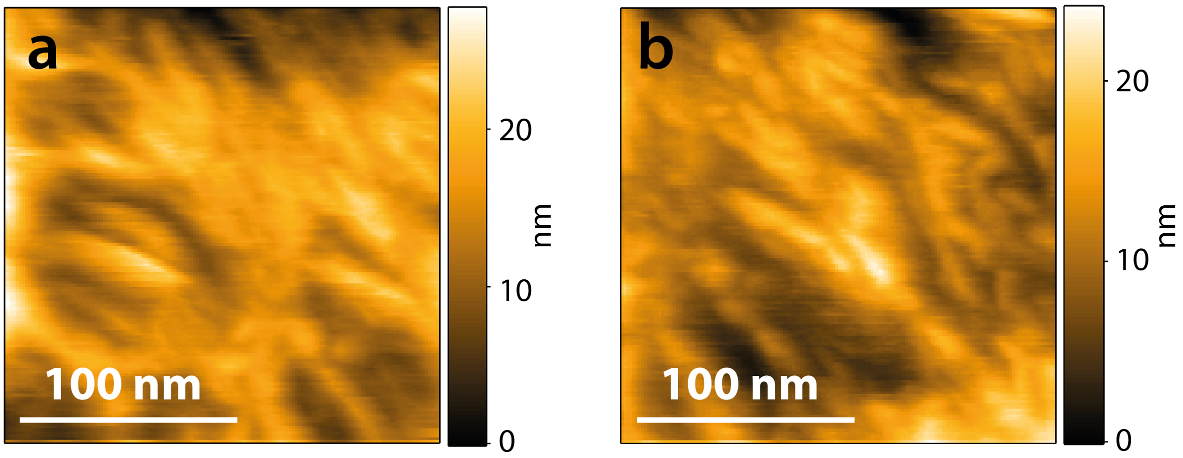

Fig. S6: AFM topography images of a) amorphous cellulose obtained from regenerating trimethylsilyl cellulose on mica in SPB and b) xylan in solution (10 g L^{-1}) added to the buffer for adsorption to the cellulose in SPB.
